# Virtual reality perspective-taking increases cognitive empathy for specific others

**DOI:** 10.1371/journal.pone.0202442

**Published:** 2018-08-30

**Authors:** Austin van Loon, Jeremy Bailenson, Jamil Zaki, Joshua Bostick, Robb Willer

**Affiliations:** 1 Sociology Department, Stanford University, Stanford, California, United States of America; 2 Communications Department, Stanford University, Stanford, California, United States of America; 3 Psychology Department, Stanford University, Stanford, California, United States of America; University of Vienna, AUSTRIA

## Abstract

Previous research shows that virtual reality perspective-taking experiences (VRPT) can increase prosocial behavior toward others. We extend this research by exploring whether this effect of VRPT is driven by increased empathy and whether the effect extends to ostensibly real-stakes behavioral games. In a pre-registered laboratory experiment (*N* = 180), participants interacted with an ostensible partner (a student from the same university as them) on a series of real-stakes economic games after (a) taking the perspective of the partner in a virtual reality, “day-in-the-life” simulation, (b) taking the perspective of a different person in a “day-in-the-life” simulation, or (c) doing a neutral activity in a virtual environment. The VRPT experience successfully increased participants’ subsequent propensity to take the perspective of their partner (a facet of empathy), but only if the partner was the same person whose perspective participants assumed in the virtual reality simulation. Further, this effect of VRPT on perspective-taking was moderated by participants’ reported feeling of immersion in the virtual environment. However, we found no effects of VRPT experience on behavior in the economic games.

## Introduction

Can virtual reality be used to increase other-regarding sentiments and behaviors such as empathy and prosocial behavior? If so, through what mechanism does such an effect operate? Previous literature suggests that virtual reality can be used to increase prosocial behavior, and empathy may be the mechanism of interest. To test this, we present here the results of a three-condition pre-registered laboratory experiment (pre-registration viewable online at: https://osf.io/egq79/) in which we test the effectiveness of virtual reality perspective-taking (hereafter VRPT), or the use of immersive virtual environments to take on the perspective of someone else. We predicted that VRPT would increase the prosocial behavior of individuals as measured through a series of behavioral games. We measure empathic concern and propensity to take on the perspective of others at time-one (before the experimental session) and at time-two (after the intervention, before the prosociality measures) to test for empathy as a mechanism.

We hypothesize, in accordance with previous literature, that VRPT will increase prosocial behavior, here measured as decisions in behavioral games. More specifically, we hypothesize that individuals who take the perspective of an individual and then interact with that same individual (i.e. those in the “direct empathy” condition) will have increased prosocial behavior when compared to those who don’t take on the perspective of anyone and then interact with someone (i.e. those in the “control condition”), and that individuals who take the perspective of someone and then interact with someone else (i.e. those in the “indirect empathy” condition) will have levels of prosociality between these two other groups. Additionally, we hypothesize that VRPT will increase both empathic concern (emotional empathy) and perspective taking (cognitive empathy) towards future interactants, and that this increase in empathy will mediate the hypothesized increase in prosocial behavior. Lastly, we hypothesize that this increase in empathy will be moderated by one’s reported “presence” in the immersive virtual environment, or how much the participant responded feeling as if they were “really in” in the virtual environment.

### Previous work

#### Empathy and prosociality

Empathy, *the ability and tendency to share and understand others’ internal states* [1], comprises multiple psychological components [2]. Experience sharing is *the tendency of an individual to vicariously take on the sensorimotor*, *visceral*, *and affective states of another* [3–5]. Experience sharing is closely related to the process of self-other merging: *the process by which another comes to seem more “self-like”* ([6–9], cf. [10–11]). Mentalizing or perspective-taking is *the act of attributing mental states to others and reasoning about how situations relate to them (e*.*g*. *cause them)* [12]. Lastly, empathic concern is *an affectively-tinged motivation to increase the welfare of another individual* [1].

These multiple empathic “pieces” feed into prosocial actions. For instance, experience sharing—indexed both through reports of self-other overlap and brain “mirroring” of affective states—predicts prosocial behavior [13–15]. However, sharing others’ distress can also motivate avoidance behavior and thus inhibit helping behavior [16–17]. Likewise, encouraging participants to mentalize about a target increases their prosocial behavior towards them [18–19] and mentalizing-related brain activity predicts later prosocial behavior [20].

People are relatively stable in their levels of empathy [21–22], but situations also powerfully affect levels of empathy by triggering or inhibiting empathic responses [23–25]. Thus, empathy (and therefore prosocial behavior) can be purposefully increased and decreased, much like a muscle which one can work to increase its strength. The goal of this paper is to further the research on an “exercise” for the “muscle” that is empathy, which, in doing so, increases an individual’s capacity to be prosocial.

#### Perspective-taking interventions

The “muscle” of empathy can be worked through perspective-taking exercises, which can generally be broken into two categories: *experiential* and *expressive* interventions [26]. The intervention we present here is of the former category, which “*feature tasks that encourage ‘tuning in’ to targets’ internal states*” ([26], pp. 206). Other such exercises in the literature include asking female college students to imagine the daily lives of and feelings of various stigmatized groups, such as AIDS patients and homeless persons to promote more positive attitudes towards them [19], having able-bodied college students travel around their campus in a wheel chair to create more positive attitudes towards the disabled individuals [27], and having Arab students reading a letter from a Jewish mother to their child to decrease hostility towards Israelis [28].

For some interventions, increases in self-reported empathy measures is the primary outcome of interest (e.g. [28–30]). For other interventions, prosocial behavior is increased towards the target group or individual [31–32]. Few studies look at both increases in empathy *and* behavioral measures, and especially so in looking for behavioral changes as being possibly mediated by increases in empathy, though such a mechanism is often implicitly posited. Here we seek to explicate and test for this.

#### Virtual reality perspective-taking

More recently, various researchers have examined the possibility of moving these perspective-taking exercises into virtual reality. More specifically, it has been examined whether traditional role-playing exercises can be enhanced or at least replicated through the use of computer generated, 3D environments in which individuals can interactively experience while “in” virtual reality, also known as IVEs. The use of IVEs to have an individual take on the perspective of another individual is known as virtual-reality perspective-taking (VRPT). Moving these exercises to virtual reality will allow social psychologists to overcome various methodological problems including the “the experimental control–mundane realism trade-off, lack of replication, and unrepresentative sampling” ([33], pp. 103).

If perspective-taking exercises are moved into virtual reality, then experiences can be induced which would otherwise be prohibitively difficult or impossible. For instance, in virtual reality researchers can have individuals experience having a different skin tone [34], age [35], or gender [36], or to even inhabit the body of an animal [37] relatively easily while maintaining tight experimental control. While other research has come up with ways to use other forms of media for perspective-taking exercises [29–30], some research suggests that IVEs can have a stronger impact on attitudes of individuals when compared to other forms of non-immersive media [38]. VRPT also allows for researchers to have fine-grained control over the experiences of participants. For instance, Mel Slater and colleagues put participants into exactly identical scenarios where they observed another individual in need of help, but with one exception: the individual in need either looked at them or did not [39]. Here we can see one of the benefits of VRPT; researchers not using virtual reality would have trouble making a tightly-controlled and/or realistic simulation of this experience for participants.

Researchers have found success in using VRPT to induce helping behavior [40], reduce implicit bias [41–43], reverse racial in-group bias [44], enhance financial planning [45], and decrease prejudice [35]. While some researchers have posited possible mechanisms for how VRPT might affect perceptions of “outgroups” [46], rigorous evidence has yet to be brought to bear on the question of what the exact social-psychological mechanism is through which VRPT exercises induce helping behavior. We believe this mechanism is empathy. Other research supports the claim that virtual reality can increase empathic response [47] and other facets of empathy [48], and analyses from previous research [40] show that this intervention is more impactful for those who had low levels of empathy prior to the intervention. However, direct evidence that empathy mediates the relationship between virtual reality use and increased cooperation is lacking. In the present study, we test specifically for increases in empathy as a mechanism through which VRPT increases prosocial behavior.

Specifically, we expect VRPT to increase both perspective taking and empathic concern. Since IVEs are more immersive than other stimuli such as imagined situations or standard two-dimensional videos, the stimuli are more immediate to individuals and can have more of an impact on their attitudes [39]. We expect the exercise to make considering the situation of the target less cognitively taxing, thus increasing on average perspective-taking, which may then lead to an increase the motivation for altruism, or empathic concern [1]. We measure both of these directly, as well as prosocial behavior through standard economic games.

## Materials and methods

### Participants

A sample of undergraduate students was recruited from a medium-sized private university on the west coast. Most participants received $100 for their participation, though small portion (n = 35) were given extra credit for participation and not paid. Including an indicator variable for whether the participant was paid or received extra credit did not qualitatively affect our results. After dropping participants who were suspicious of the experiment (more on this below), the sample consisted of 180 participants (N = 180): 72 males, 106 females, and 2 individuals who identified as some other gender. The total sample ranged in age from 18 to 29 (M = 20.28) and was racially diverse. See Table 1 below for demographics of participants by condition.

**Table 1:** Demographics by condition.

	Control				Direct Empathy				Indirect Empathy			
	M	F	O	T	M	F	O	T	M	F	O	T
Am. Ind. or AK Native	0	0	0	**0**	0	1	0	**1**	0	1	0	**1**
Asian	2	6	0	**8**	7	9	0	**16**	6	9	0	**15**
Black or African-American	7	2	0	**9**	3	1	1	**5**	6	4	0	**10**
HI N & Pacific Islander	1	0	0	**1**	0	0	0	**0**	0	1	0	**1**
Latino	5	6	0	**11**	3	7	0	**10**	3	5	0	**8**
White	11	19	0	**30**	6	11	0	**17**	11	17	0	**28**
Some Other	1	3	0	**4**	0	3	0	**3**	0	1	1	**2**
**Total**	**27**	**36**	**0**	**63**	**19**	**32**	**1**	**52**	**26**	**38**	**1**	**65**

“M” column shows number of participants who identified as male in each condition, “F” column shows number of participants who identified as female in each condition, the “O” column shows number of participants who identified as neither male nor female in each condition, and “T” shows the total number of participants in each condition. “Latino” participants are anyone who marked any race but identified as being “Latino or Hispanic”.

### Equipment

Participants viewed the virtual world using the HTC Vive, a head-mounted display (HMD), that allows for three-dimensional, stereoscopic views of a fully immersive, digitally rendered virtual reality environment. Participants also used two handheld HTC controllers to interact with objects in the virtual environment. Both the HMD and the hand controllers are tracked by two HTC Lighthouse base stations, which send out an array of non-visible light that can be detected by the HMD and hand controllers. The Lighthouse uses the light detection information to determine the 3D position of the HMD and hand controllers, as well as the orientation (pitch, yaw, and roll) of both. The 3D position and orientation of the participant’s head and hands are used to update the rendering of the first-person viewpoint accordingly. Haptic feedback in the form of vibrations through the hand controllers was generated when participants interacted with objects to increase immersion in the virtual environment.

### Procedure

The methods laid out in this article were approved by the Stanford Institutional Review Board. All participants were asked to remotely complete a pretest survey through a Qualtrics survey in which they gave informed consent to participate in the study, completed a battery of survey measures, provided basic demographic information, described their daily routine, and provided details about their personal lifestyle choices (e.g., preferences in clothing, music, art, etc.) under the guise that a virtual experience of their life might be created based on their responses for other students to experience. In fact, three IVEs were created independent of any participant’s responses, but we wanted participants to believe a virtual simulation of their life could have been created and that other people may experience theirs. This leant legitimacy to our later claims that they were experiencing a virtual environment based off the life of another student from the same university. Upon completing the survey, participants were able to sign up to participate in the second part of the study, to take place at least one week after the pretest was completed.

Upon arriving in-person for the second part of the study, participants were randomly assigned to one of the three virtual reality experiences. In the control condition, participants walked around and observed a virtual representation of the lab room where they were participating in the study. They did not have a self-avatar representation or the ability to interact with objects in the virtual environment. Participants in the other two conditions (see below) experienced a “day in the life” of a student who supposedly attended the same university as the participant, taking their perspective within the IVE by embodying a self-avatar that they were told was based off this other student. Participants in either of these conditions had an equal likelihood of embodying “Steve,” or “James” two fictional characters (with similar but not identical backgrounds) the participants were led to believe were real fellow students. This variation in stimuli was introduced to test whether increased empathy from VRPT is target-specific or generalizable to relatively similar others (more on this below).

After completing the IVE treatment, participants were led to a computer in a private survey room to answer a questionnaire and complete a series of behavioral games programmed in Qualtrics. In this portion of the experiment, they were told their answers would be “paired with another student’s”. This language is intentionally neutral so that neither competitive nor cooperative undertones are communicated to the participant, as this would likely affect behavior and perceptions of the “other student”. Thus, participants were assigned randomly to be “paired with” either “Steve” or “James”, independent of their assignment to embodying “Steve” or “James” or to the control condition. This created six possible experiences for the subject, which we group into three conditions:

1. indirect empathy (*n* = 65): the participant embodies “James” (“Steve”) and then is “paired” with “Steve” (“James”). We hypothesize that this condition will induce the second most empathy and will therefore elicit the second most prosocial behavior.
2. direct empathy (*n* = 52): the participant embodies “James” (“Steve”) and then is paired with “James” (“Steve”). We hypothesize that this condition will induce the most empathy and will therefore elicit the most prosocial behavior.
3. control (*n* = 63): the participant embodied no one (and walked around a virtual version of the lab room), and then was paired with either “James” or “Steve”. We hypothesize that this condition will induce the least empathy and will therefore elicit the least prosocial behavior.

Results are robust to specification of “condition” as either direct empathy, indirect empathy, and control, or as the six possible experiences (takes the perspective of Steve and interacts with James, takes the perspective of James and interacts with James, etc.) the participant may have been exposed to, but the former is preferred for interpretability.

### Design

**Fig 1:**
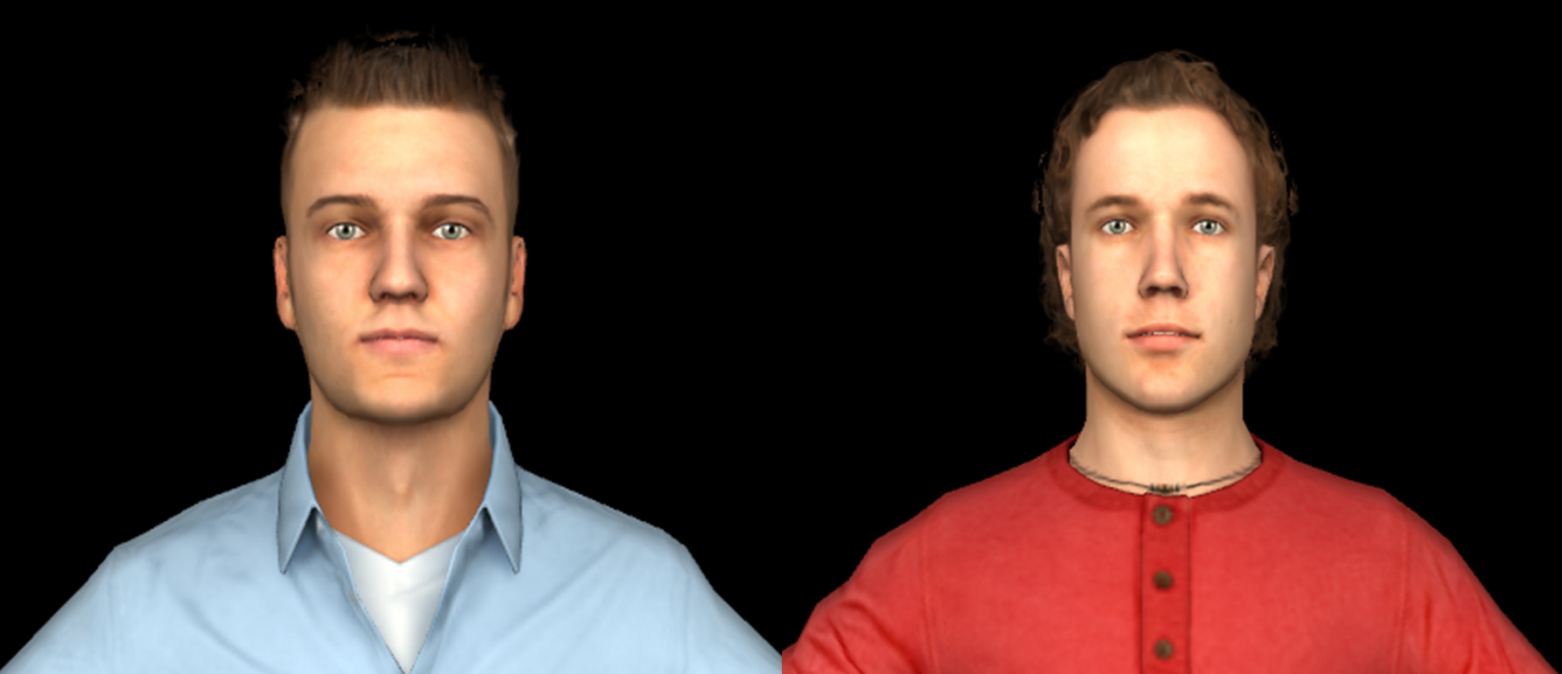
Avatars. The participants may have taken the perspective of James (left) OR Steve (right).

In the IVEs created for the experimental conditions, the narrative began with participants standing in an undergraduate dorm room (designed to look like the actual dorm rooms of the university “Steve” or “James” was supposedly from), facing a mirror in front of a sink. Participants were introduced to their self-avatar, which they saw either as an avatar named Steve or an avatar named James (Fig 1). They were given a few moments to adjust to their virtual environment, after which they were prompted to perform a series of simple movements in front of the mirror to help them associate their body’s physical movements with the avatar’s movements that they saw in the mirror. This helped invoke “body transfer”.

**Fig 2:**
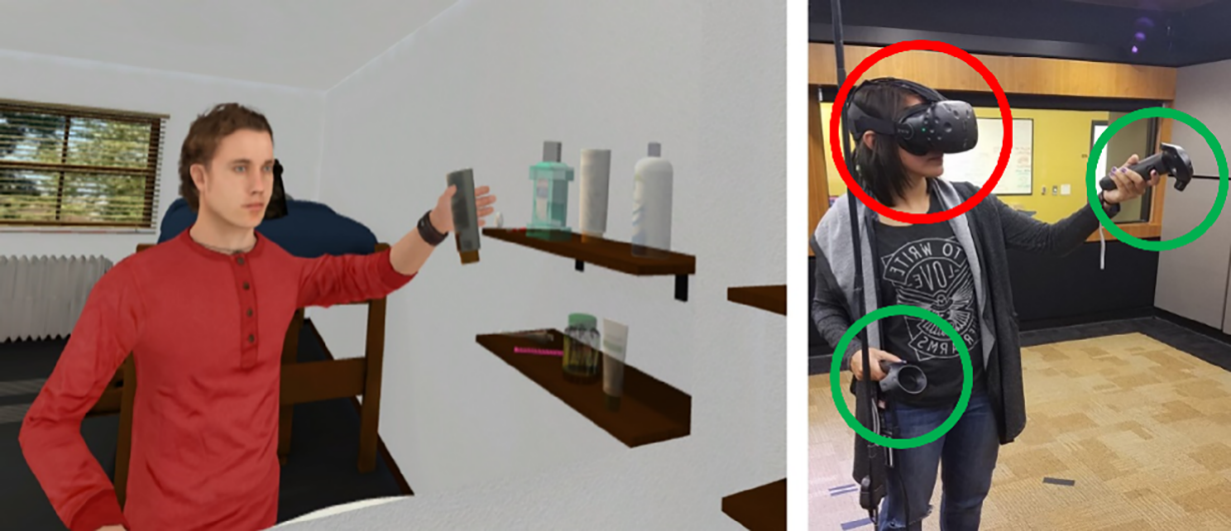
Equipment/first task. Participants hold the HTC controllers (indicated with green circles) in their hands and wear the HTC Vive HMD (indicated with a red circle) on their head as shown and interact with the IVE as their real-world sensory input is replaced with the world of the IVE. The individual in this image (as well as in Figs 3 and 4) has given written informed consent (as outlined in PLOS consent form) to publish these photos.

**Fig 3:**
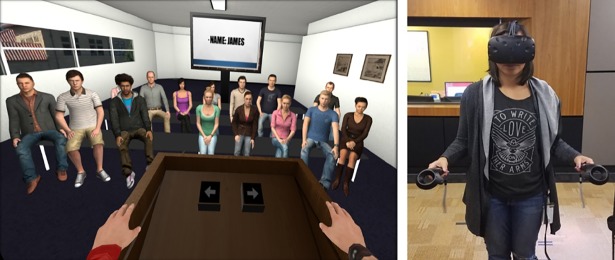
Second task. Participants stand at a podium and give a presentation about “themselves” based on information presented to them via a screen in the back of the classroom.

**Fig 4:**
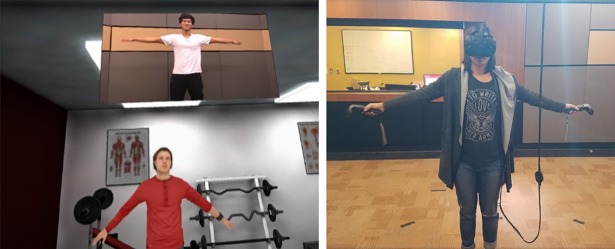
Third task. Participants complete a series of exercises while looking at a screen placed such that they also see a reflection of their avatar.

After becoming acquainted with the virtual environment, participants “unpacked their suitcase” by picking up objects with their hand controllers and placing them in their designated spots on shelves in the room (Fig 2). After completing this task, participants were instructed that they were about to “attend class”. Participants were then transported to a classroom, where they stood behind a podium and were instructed to give a presentation to a virtual class seated in front of them (Fig 3), based off presentation slides which contained information in bullet point format about either Steve or James (whichever environment they were assigned). They were instructed to speak as if they were Steve or James and elaborate on each bullet point. The authors felt that giving participants sparse information (bullet points) and asking them to expand that information into full, natural-language sentences was the best combination of maintaining experimental control and forcing participants to engage with the information presented. Having participants recite fully-defined text would lead to a lack of cognitive engagement, but having the participants imagine information from nothing would likely lead to huge individual variation and add “noise” to the results. After completing their presentation, participants went to “work out” at a small gym. Here, participants were asked to follow along with a workout video, performing stretches and arm movements in front of a mirror (Fig 4).

### Pre-manipulation (time-one) independent variables

Interpersonal Reactivity Index (IRI) [49] -Three items on a five-point interval scale were selected because of the large number of questions present on the time-one survey from two sub-scales of the IRI that measure tendency towards empathic concern (EC; experience sharing) and perspective taking (PT; mentalizing), making a total of 6 items. A sample item from the EC subscale is “I would describe myself as a pretty soft-hearted person” and a sample from the PT subscale is “I believe that there are two sides to every question and try to look at them both” (1 = Does not describe me well, 5 = Describes me well; Cronbach’s α for all items = .68, for PT items = .73, for EC items = .74).

Social Value Orientation (SVO) [50]—a nine-item questionnaire which presents participants with three possible outcomes, of which they select their most preferred. Each of these three possible outcomes is indicative of either a competitive orientation, an altruistic orientation, or a prosocial orientation. If a participant answered consistently (6 or more times) in one style of orientation, they were considered to be of that orientation.

Philosophies of Human Nature and Trustworthiness (abbreviated from [51])—A six-item questionnaire to assess tendency to trust others and belief in the good of human nature. Participants were instructed to read each prompt and then use a slider (0 = Disagree, 100 = Agree), which reflects their first impression and views of human nature (M = 49.9, SD = 9.2).

NEO-Altruism [52]—We employed an eight-item, five-point interval scale questionnaire drawn from the NEO-inventory to measure how altruistically participants thought others viewed them to be and how altruistically they viewed themselves. Sample items were, “I’m not known for my generosity” (reverse-coded) and “I try to be courteous to everyone I meet” (1 = Strongly Disagree, 5 = Strongly Agree; Cronbach’s α = .66).

### Post-manipulation (time-two) independent variables

Inclusion of Other in Self (IOS) [53]—This measure was adapted and employed to measure how connected participants who either embodied James or Steve (i.e. participants who were not in the control condition) felt with the avatar that they embodied. This is a single-item, seven-point scale depicting a series of increasingly overlapping circles (similar to a Venn diagram), one circle labeled “self” and one circle labeled “James” or “Steve,” depending on who they embodied during the treatment. Participants chose the overlapping circles they felt best represented how connected they were with James or Steve.

Body Transfer [37]–An eleven-item, seven-point interval scale assessed how much the participant felt as if they had become James or Steve in the direct empathy and indirect empathy conditions. Sample items were, “In the virtual environment, how much did you feel that your avatar’s body was your body” and “When you were looking in the mirror how much did you feel a strong connection with your avatar as if you were looking at yourself” (0 = Not at All, 10 = Very Much; Cronbach’s α = .90).

Spatial Presence [54]—A five-item, five-point interval scale was used to measure perceived spatial presence. Sample items were, “To what extent did you feel that you were really inside the environment?” and “To what extent did you feel that you were surrounded by the environment?” (1 = Not at all, 5 = Very strongly; Cronbach’s α = .77).

Positive and Negative Affect Scale (PANAS) [55]—A 20-item, five-point interval scale was employed to measure participants’ positive and negative affect at the time of their post-manipulation survey. We chose to add two prompts to the questionnaire to explore gender, “Masculine” and “Feminine”, making a total of 22 items. The initial prompt informed participants that they would need to, “Indicate to what extent you feel this way right now, that is, at the present moment.” Sample prompts were “Alert” and “Hostile”. (1 = Very slightly or not at all, 5 = Extremely; Cronbach’s α = .84)

### Dependent variables

Post-Manipulation (Time-two) Perspective-Taking—We adapt from the perspective taking subset of the IRI and employ a three-item, five-point interval scale to measure, after being “introduced” to the other student whose answers will be paired with theirs (either “James” or “Steve”) via a short paragraph and picture, the participant’s propensity to take the perspective of this other student. Sample items include asking how much the participant is “…making an effort to see the world through [James’ or Steve’s] eyes” and “…imagining how [James or Steve] is feeling.” (1 = Does not Describe Me Well, 5 = Describes Me Well; Cronbach’s α = .92)

Post-Manipulation (Time-two) Empathic Concern*—*We adapt from the empathic concern subset of the IRI and employ a four-item, five-point interval scale to measure, after being “introduced” to the student whose answers will be paired with theirs (either “James” or “Steve”) via a short paragraph and picture, the participant’s sense of empathic concern towards their future partner. This scale asks how much the participant is feeling “Sympathetic” and “Compassionate” towards James/Steve upon learning they will be interacting with them (1 = Does not Describe Me Well, 5 = Describes Me Well; Cronbach’s α = .71). Given the relatively low alpha score of this scale, we conducted principal component analysis on the four different measures. The first component had an eigenvalue of 2.18 and explained 54 percent of the variance of the scale. Each item had a similar weighting onto this component (weights of 0.48, 0.48, 0.51, and 0.52). The second component had an eigenvalue of 1.07 and explained 27 percent of the variance. We estimated the first component and replicate all analyses presented here with qualitatively similar results. There was no single item which increased the alpha score of this scale if dropped.

Trust Game [56]—In this behavioral game, one individual (the “first mover”) is given an allotment of currency (in our case, 10 “lab dollars”, which participants believed would be exchanged for real currency at the end of the study), any amount of which they can choose to entrust to the “second mover”. This entrusted amount is tripled, and the second mover can choose to return as much or as little of this tripled amount they like back to the first mover. If both players act cooperatively, both can end up with 15 lab dollars (the first mover entrusts all 10 lab dollars, which is tripled to be 30 dollars, which the second mover divides evenly), but if the first mover doesn’t trust the second mover (and entrusts less or none of the original endowment), or if the second mover betrays the trust of the first mover and keeps more than their “fair share” of the tripled endowment, then this (in some ways) optimal outcome cannot be reached.

Participants were instructed on how the game works and were asked a series of questions to make sure they understood the game sufficiently. They then played two rounds of the game, the first as the first mover, where the second mover was the “other student”, and the second as the second mover after the “other student” (being the first mover in this round) had entrusted them with all 10 lab dollars (Participants were told the “other student” had performed these tasks prior to the participant, and their answers were being paired after-the-fact). The amount they entrust to the “other student” as the first mover is indicative of trust towards them (M = 5.9, SD = 3.2), while the amount they return as the second mover measures how much greed they display towards the “other student” (M = 11.8, SD = 5.9).

Dictator Game [57]—In this behavioral game, the Dictator, known as the “Decider”, would receive an endowment of 10 lab dollars and decide how many lab dollars would be sent to the “Receiver”, after which the task would be complete; there was no recourse for the Receiver and they would have to accept whatever amount the Decider sent. The participant played one round of this game as the “Decider”, which is an additional way to measure levels of greed the participant displays towards the “other student” (M = 3.2, SD = 2.2).

**Fig 5:**
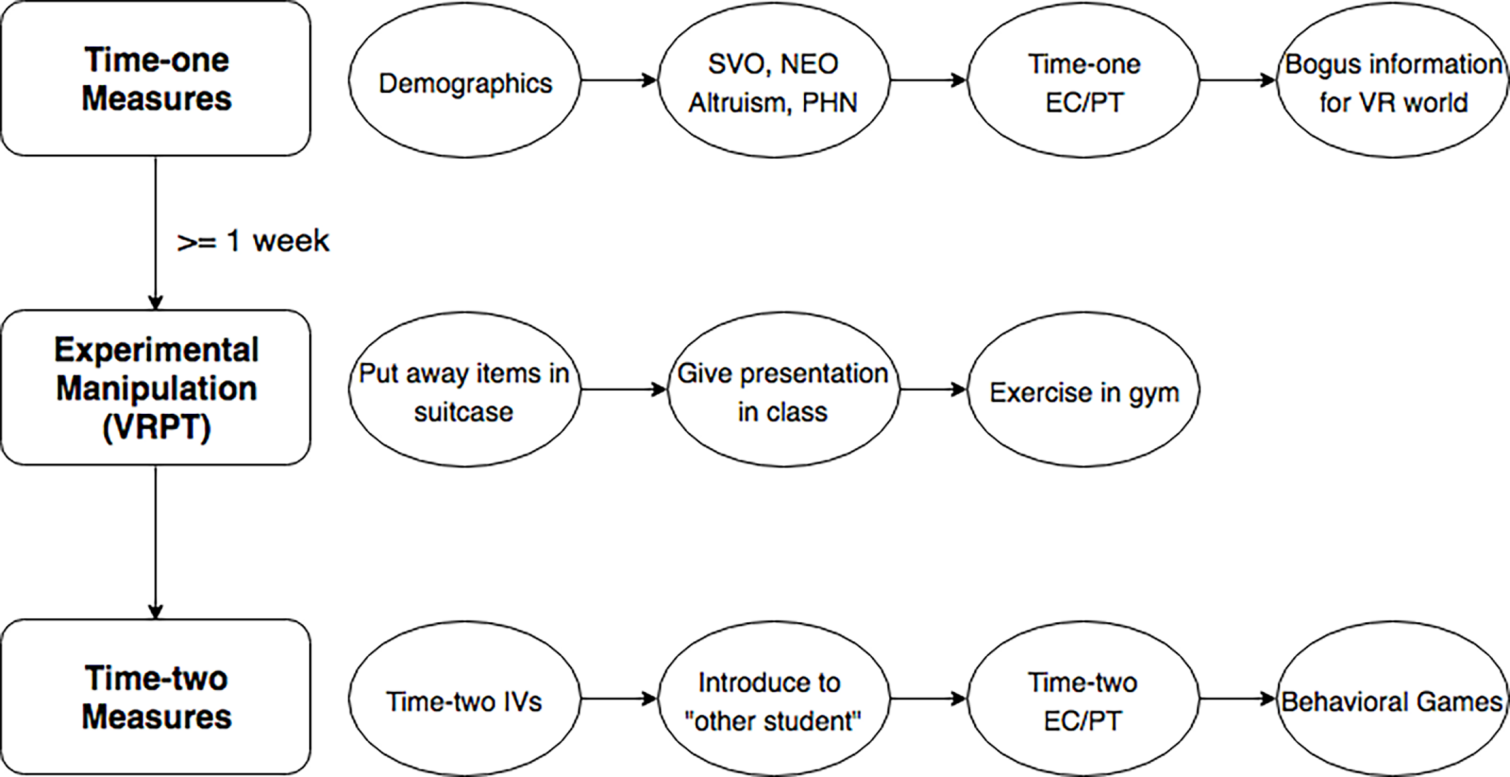
Order of experimental materials.

Circle Tracking Game—As part of the exploratory aspect of this study, we developed a computer mediated coordination game, the circle tracking game (CTG), to investigate if there were any differences in participant performance based on their randomly assigned condition (control, direct empathy, and indirect empathy). The CTG consisted of two distinct elements, a ball and a participant-controlled hoop. Participants believed that the ball was a recording of mouse movements the “other participant” had recorded when they completed this task at an earlier time. In reality, this was one of several recorded mouse movements of a member of the research team. Participants’ role in this game was to use their computer mouse to control the hoop and to keep the ball within the boundary of the hoop for as long as possible during the one-minute task. We measure the amount of time they successfully keep the moving ball within the hoop. See below (Fig 5) for a graphic depicting the order of the collection of variables.

### Analytic strategy

To test our hypotheses, we use ordinary least squares (OLS) regression [58]. To see if there are differences in a self-report or behavioral measures across conditions, we regress this dependent variable on a set of two indicator (binary) variables, one of which is equal to one if the participant is in the direct empathy condition and zero if not, and the other which is equal to one if the participant is in the indirect empathy condition and zero if not. The coefficients in the regression associated with these variables can be interpreted as the mean difference between the respective condition and participants in the control condition. For presentation purposes, to test for differences between the two experimental conditions, we prefer a linear combination or linear contrast post-estimation test to running a separate model with one of the experimental conditions as the withheld group.

To “control” for other variables (examine the effect of the manipulations as independent linear contributions to the dependent variables to the “controlled for” variables), we simply include these variables in the list of regressors for the regression. To test for “moderation” (whether the effect of one regressor on the dependent variable is contingent on the value of another regressor), we include both regressors as well as the product of the two regressors in the regression. We use a standard *α* = 0.05 threshold for reporting significance, but report either standard errors or p-values for all meaningful coefficients associated with a statistical test. We elect, however, to not report these for coefficients associated with “constants” (expected value of the dependent variable when all regressors equal zero) for presentation purposes. For further details on other analyses, see our pre-registration form at the link in the introduction section.

## Results

We pre-registered many features of the current experiment online at the Open Science Framework (OSF), including a detailed explanation of randomization and experimental procedures, intended analyses, all measured variables, criteria for dropping participants from analyses, and predicted findings (find the link to our pre-registration in the introduction section of the paper). In this section we report the results from the pre-registered analyses for the experiment. At the end of the experimental session, participants filled out two open-ended questions concerning their guesses regarding the hypotheses of the experiment and any additional thoughts on the experiment. We coded each of these open-ended responses for levels of reported suspicion regarding aspects of deception in the study. Eight individuals were found to be suspicious enough to be removed from analysis (these eight individuals doubted the existence of an actual student corresponding to the avatar James or Steve; their inclusion does not affect the results reported here).

Table 2 and Table 3 present results of OLS linear regression models analyzing the effect of experimental condition on our dependent variables. Table 2 shows the effect of condition on the various self-reported survey questions, while Table 3 shows the effects of condition on our behavioral measures, specifically the cooperation games and the circle-tracking game (CTG). Means and standard deviations for behavioral games can be seen in S1 and S2 Tables. As indicated by all models in Tables 2 and 3, being in the indirect empathy condition (the condition in which a participant either took the perspective of James and then interacted with Steve or took the perspective of Steve and then interacted with James) did not have a significant effect on any of our dependent measures in comparison to the control group (those who did not take the perspective of James or Steve before interacting with either James or Steve).

**Table 2:** Effect of condition on self-report measures at time 2.

	*Time-2* *Perspective Taking*	*Time-2* *Empathic Concern*	*Spatial Presence*
Condition *(Control as constant)*			
*Direct Empathy*	0.924* (0.42)	-0.097 (0.28)	-0.264* (0.12)
*Indirect Empathy*	0.175 (0.39)	0.12 (0.27)	-0.166 (0.11)
Constant	4.974	2.472	3.679
R^2^	0.030	0.001	0.028
N	180	180	180

* p < 0.05

** p < 0.01

*** p < 0.001

All results are *β/SE*; Significance symbols and standard errors on constants are excluded

**Table 3:** Effect of condition on behavioral measures.

	*Dictator Game*	*Trust Game* *(1*^*st*^ *Mover)*	*Trust Game* *(2*^*nd*^ *Mover)*	*Circle Tracking Game (sec)*
Condition *(Control as constant)*				
*Direct Empathy*	-0.456 (0.40)	-0.192 (0.60)	0.162 (1.10)	-0.574 (.89)
*Indirect Empathy*	-0.648 (0.38)	-0.123 (0.57)	-0.257 (1.04)	-0.095 (.838)
Constant	3.571	6.000	11.857	43.407
R^2^	0.017	0.001	0.001	0.003
N	180	180	180	180

* p < 0.05

** p < 0.01

*** p < 0.001

All results are *β/SE*; Significance symbols and standard errors on constants are excluded

**Fig 6:**
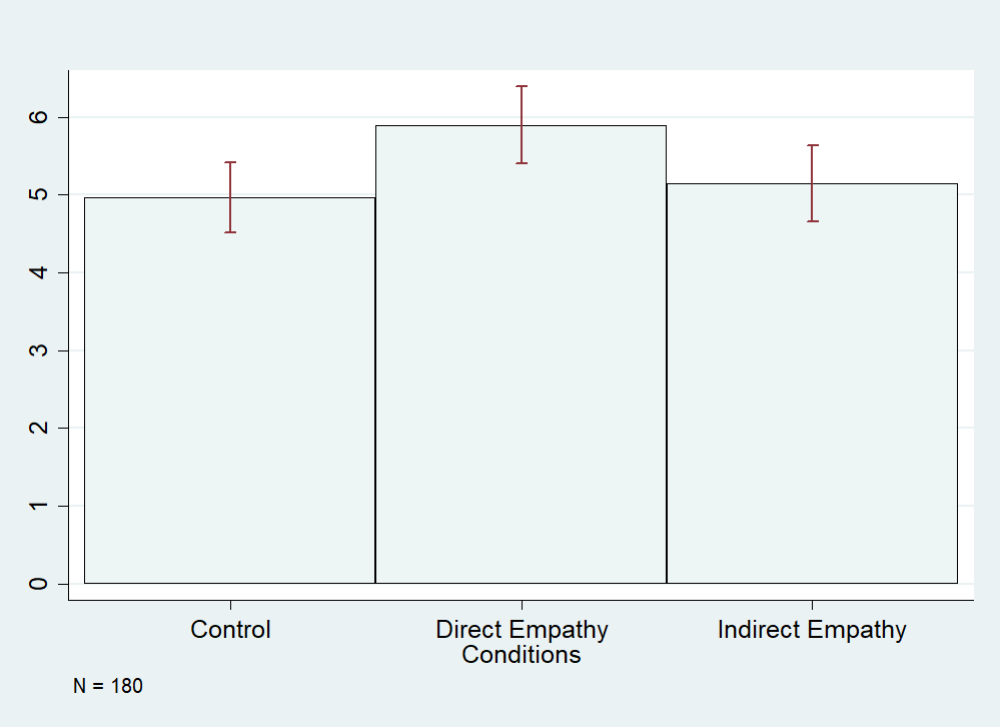
Effect of condition on time-2 perspective taking.

As shown in Table 2 Model 1, being in the direct empathy condition (the condition in which the participant took the perspective of the same person they interacted with) did have a significant effect on participants’ self-reported propensity to take the perspective of the partner assigned in part 2 of the study, following the virtual reality experience (Fig 6). That is, participants in this condition reported trying to take the perspective of their interaction partner more so than those in the control condition, and this difference was statistically significant (*β* = .924, *p* = .028). Using a linear combination post-estimation test, we find that the comparison between the indirect and direct empathy conditions is only marginally significant (*β* = .749, *p* = .072). Those in the direct empathy condition did not cooperate more than those in the control condition in any of the behavioral games, nor did they perform better on the circle tracking game (CTG). Additionally, those in the direct empathy condition felt less presence in their respective IVE than those in the control condition. This finding, in retrospect, isn’t surprising: participants would likely feel they are “more in” a virtual simulation modeled to look just like the lab room they are actually in than a simulation of various digital rooms they have possibly never seen before.

To make sure that potential failures of random assignment do not explain the perspective-taking finding, we conducted a multivariate regression analysis statistically controlling for gender, race, time-one perspective-taking propensity as measured by the perspective-taking facet of the IRI, NEO-Altruism Scale score, social value orientation, and Philosophy of Human Nature and Trustworthiness Scale score. After including these control terms, all collected from the pre-screen questionnaire, the result that those in the direct empathy condition reported more propensity to take the perspective of the individual they were interacting with in comparison to those in the control condition remained significant (*β* = .897, *p* = .036). The linear combination postestimation test for comparing the effect of the indirect and direct empathy conditions was still only marginally significant after controlling for these factors (*β* = .767, *p* = .069). Thus, we are unable to offer support to the conclusion that the direct empathy condition evoked more propensity to take an interactant’s perspective than the indirect empathy condition, though we do find that the direct empathy condition evoked significantly more propensity to take an interactant’s perspective than the control condition, even after controlling for pre-intervention propensity to take others’ perspectives, gender, SVO, NEO-Altruism score, PHN-Scale score, and race. This effect does become non-significant, however, after controlling for the individual’s PANAS score (*β* = .763, *p* = .068). All other results reported above remain non-significant after controlling for these same variables (see the S3 and S4 Tables for tables of all models).

**Fig 7:**
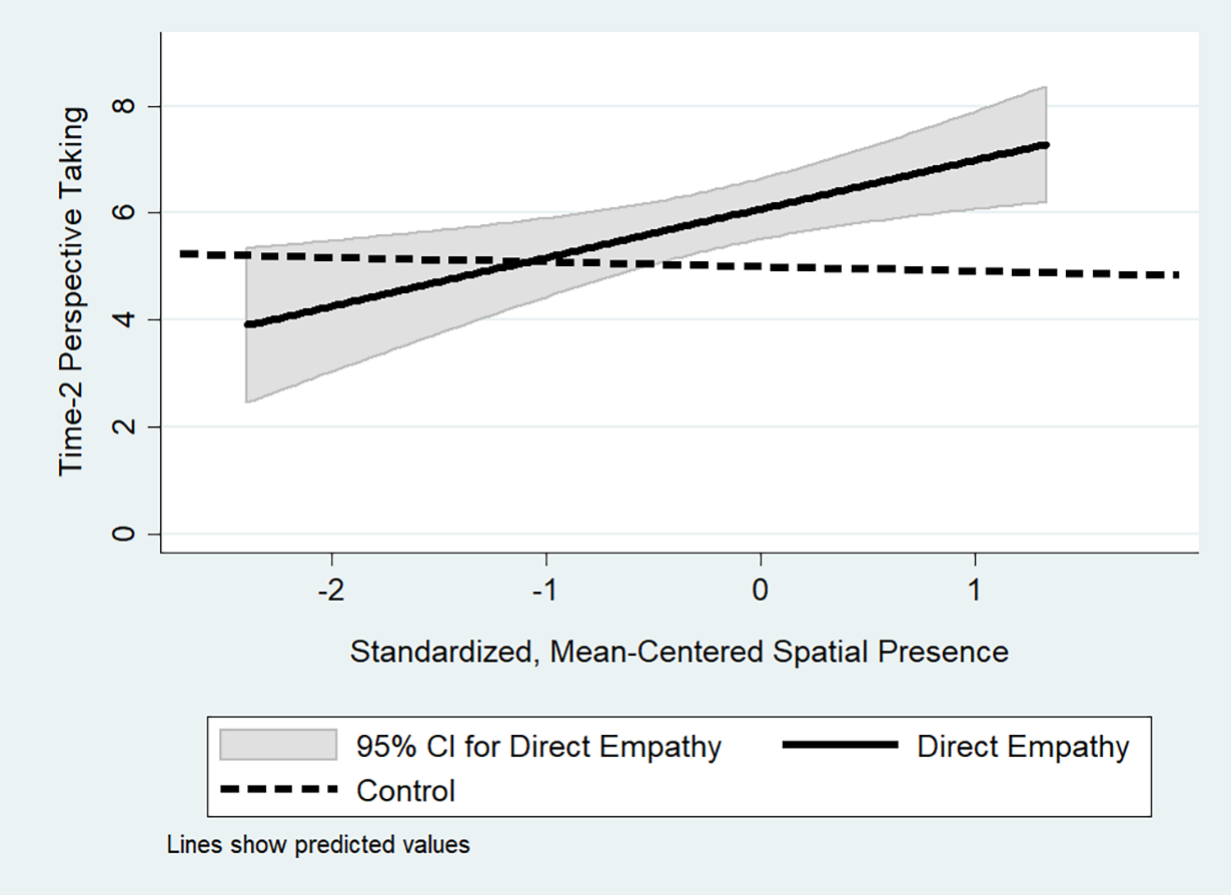
Effect of condition and presence on time-2 perspective taking.

As shown in Model 3 of Table 4, the effect of being in the direct empathy condition on time-two perspective-taking is moderated by the participant’s sense of presence in the IVE, i.e., their sense that they were actually in the virtual environment and that what was happening in the virtual environment was actually happening (how “I” they found the “IVE”; Fig 7). Three interesting things should be noted from this finding. The first is that as one’s feeling of presence in the IVE increases, so does the effect of being in the direct empathy condition on self-reported propensity to take the perspective of their future interactant. Second, this only holds for the direct empathy condition, and not the indirect empathy condition. The third interesting thing is that the size of the coefficient of the interaction effect is roughly equal in size to the coefficient of the main effect. This means that if an individual felt little presence in the IVE (more precisely, if they felt one standard deviation of presence below the mean presence reported by participants, since presence is a standardized and mean-centered variable), then the intervention had no effect on future perspective-taking. A linear combination post-estimation test confirms this last intuition (*β* = .089, *p* = .881).

**Table 4:** Effect of condition and presence on time-2 perspective taking.

	*M1*	*M2*	*M3*
Condition *(Control as constant)*			
*Direct Empathy*	0.924* (0.42)	—	1.086* (0.42)
*Indirect Empathy*	0.175 (0.39)	—	0.148 (0.39)
Presence *(Standardized and* *mean-centered)*	—	-0.060 (0.17)	-0.087 (0.28)
Presence * Condition *(Control as constant)*			
*Direct Empathy*	—	—	0.998* (0.43)
*Indirect Empathy*	—	—	-0.103 (0.38)
Constant	4.974	5.304	4.992
R^2^	0.030	0.001	0.072
N	180	180	180

* p < 0.05

** p < 0.01

*** p < 0.001

All results are *β/SE*; Significance symbols and standard errors on constants are excluded

## Discussion

We find that our VRPT exercise, under certain conditions, increased participants’ propensity to take the perspective of their partners (Fig 6). The first condition is that participants felt some minimal amount of “presence” in the IVE (Fig 7), and the second is that the VRPT exercise has the participant take the perspective of the same individual they later interact with. In other words, the VRPT exercise must be relatively immersive and target-specific. We also find that the increase in empathy towards a target we observe when an individual takes the perspective of that same target in a VRPT exercise, in this case, was not strong enough to induce an increase in prosocial behavior. We thus find support for our hypothesis that VRPT increases empathy and that this increase is moderated by presence but fail to find that VRPT increases prosocial behavior.

Increased perspective taking may be the result of the mere exposure effect [59]. That is, the observed increase in participants’ reported willingness to take on the perspective of John or Steve in the direct empathy condition (in comparison to the control condition) may simply be a result of seeing more of John or Steve, which leads them to like Steve or James more. For exploratory analyses not presented here, near the end of the survey, we asked a series of questions asking if participants would want to share emails with the other student, and how they perceived the other student (whether they believe they would enjoy spending more time with them, whether they think they were a good person, whether they were glad they were paired with them, and whether they like them). For each of these five measures, for both John and Steve, a t-test was performed to see if those in the direct empathy condition perceived their partner differently from or were differently likely to share their email than those in the control or indirect empathy condition. The only of these 10 t-tests which were significant in a two-tailed test was whether they viewed Steve as a nicer person (*p* = 0.044). When these five measures were put into an alpha scale (*α*_*James*_ *=* 0.90, *α*_*Steve*_ *=* 0.91), those in the direct empathy condition had a significantly *lower* score in a two-tailed t-tests (*β*_*James*_ = 30.92, *p*_*James*_ *=* 0.002; *β*_*Steve*_ = 31.57, *p*_*Steve*_ *=* 0.002). This is consistent with previous work [38] which finds that embodiment of VR avatars has an effect above and beyond simple priming. This last finding is inconsistent with the mere exposure effect explanation, though we cannot rule this out as an explanation of our results. Future work should more explicitly examine this as a possible mechanism for increasing empathy towards targets of VRPT.

A reasonable question concerning our results is whether it was simply the stress and/or anxiety produced by the “presenting in front of a class” exercise that caused the change in empathy that we observe [60–61], especially when one considers the results that our main finding is statistically significant in a model with all controls besides the PANAS score, but then falls below significance when the PANAS score is included. We don’t believe this is the case for a couple of reasons. First and foremost, our effect is significant when a participant’s PANAS score is the only control entered into the regression. This leads us to believe the null finding is more from a lack of statistical power. Secondly, we test whether specific items on the PANAS (‘anxious’, ‘distressed’, ‘jittery’, ‘afraid’, ‘upset’, and ‘nervous’) are significantly lower in the control condition than in the two experimental conditions. This is not the case, nor is an alpha scale of the six measures significantly different. However, with our current design we cannot rule this out as an explanation of our results.

Our study is limited in various ways. First and foremost, our key finding that VRPT increases cognitive empathy is dependent on a measure that is adapted from only part of the standard IRI. Future research should use different measures of cognitive empathy, including the full perspective-taking subset of the IRI and more systematically derived measures. Indeed, our time-one empathy measures are only part of the standard IRI and mirror this limitation. Additionally, future research should do pre-testing and use a power and/or precision analysis to more systematically derive an appropriate sample size, which we do not do here due to resource constraints and the exploratory nature of the study. Lastly, though we tried to use as neutral of language as possible in presenting the various economic games, it is unlikely that participants didn’t think of the game as competitive in some sense. Though we attempt to account for this by controlling for participants’ social value orientation, future research should consider other ways of measuring prosociality and/or pretest to determine if players consider the other interactant in the game a partner or a competitor.

Here we develop and utilize a unique paradigm to studying the effects of VRPT on interpersonal behavior. This experimental paradigm can not only be used in future research on the effects of VRPT on prosocial behavior but could also be used to study VRPT’s effects on, for instance, organizational behavior, political persuasion, and person-to-person sales. Additionally, the circle tracking game (CTG) we develop and implement here offers a novel way to study coordination amongst individuals. A .qsf file is available upon request to the corresponding author which, when imported to Qualtrics, can re-create the circle tracking game. Find the exact wording of all prompts, questions, and experimental scripts used in the lab in the supporting information for this paper.

## Conclusions

Empathy is important for positive human interaction and has been shown in previous research to be associated with prosocial behavior. VRPT is an exercise which uses virtual reality technology to get an individual to take the perspective of someone else, and in other research has been shown to increase prosocial behavior. It has been expected that an increase in empathy is the underlying mechanism for this increase in prosociality, but no previous research has rigorously demonstrated this. Here, we find that VRPT can be used to increase target-specific perspective-taking in individuals. This increase is moderated by the individual’s sense of “presence”, or how immersed in the virtual environment they report feeling. We do not demonstrate that VRPT increases prosocial behavior as measured through behavioral games.

## Supporting information

S1 Table **Appendix A.** Means and Standard Deviations of Behavioral Game Measures by Condition.
(DOCX)

S2 Table **Appendix B**. Means and standard deviations of all dependent variables.
(DOCX)

S3 Table **Appendix C.** Effect of condition on self-report measures with all controls.
(TIF)

S4 Table **Appendix D.** Effect of condition on behavioral measures with all controls.
(TIF)

S1 File Pre-test Data. A .xlsx file with the data that resulted from the time-one questionnaire described in the paper.
(XLSX)

S2 File Post-test data. A .xlsx file with the data that resulted from the time-two experiment described in the paper.
(XLSX)

S3 File CTG data. A .xlsx file with the CTG data described in the paper.
(XLSX)

S4 File Experimenter script. The script used by the researchers when being ran through the experiment.
(DOCX)

S5 File Qualtrics file (post-test). A file that can be uploaded to Qualtrics to exactly re-create the questionnaire used in the post-treatment experiment described in the paper.
(QSF)

S6 File Qualtrics file (pre-screen). A file that can be uploaded to Qualtrics to exactly re-create the questionnaire used as the pre-screen described in the paper.
(QSF)

S7 File STATA replication package (cleaning). A file that will clean data in S1 File, S2 File, and S3 File to where it can be used in S8 File in STATA 14.
(DO) Click here for additional data file.

S8 File STATA replication package (analysis). A file that will replicate all analyses, tables, and graphs shown in the paper in STATA 14.
(DO) Click here for additional data file.
