# Supplementary material for: Virtual reality perspective-taking increases cognitive empathy for specific others
Source: PLoS One. 2018 Aug 30;13(8):e0202442. doi: 10.1371/journal.pone.0202442 (PMC6116942; doi:10.1371/journal.pone.0202442)
Supplement: S1 Table — Appendix A. Means and Standard Deviations of Behavioral Game Measures by Condition. (DOCX) [file pone.0202442.s001.docx]

| **Appendix A. Means and Standard Deviations of Behavioral Game Measures by Condition** | | | | |
| --- | --- | --- | --- | --- |
|  | Direct Empathy | Indirect Empathy | Control | Max/(Min) |
| Dictator Game  Contributions | 3.12  (1.93) | 3.57  (2.13) | 2.92  (2.34) | 0  (10) |
| Trust-Game 1^st^ Mover  Contributions | 5.81  (3.06) | 6  (3.16) | 5.88  (3.35) | 0  (10) |
| Trust Game 2^nd^ Mover  Contributions | 12.02  (6.15) | 11.86  (4.94) | 11.6  (6.49) | 0  (20) |
| N | 52 | 63 | 65 | -- |
| Mean/(SD) is displayed in Direct Empathy, Indirect Empathy, and Control columns | | | | |
