# Supplementary material for: Virtual reality perspective-taking increases cognitive empathy for specific others
Source: PLoS One. 2018 Aug 30;13(8):e0202442. doi: 10.1371/journal.pone.0202442 (PMC6116942; doi:10.1371/journal.pone.0202442)
Supplement: S2 Table — Appendix B. Means and standard deviations of all dependent variables. (DOCX) [file pone.0202442.s002.docx]

| **Appendix D. Means and Standard Deviations of all Dependent Variables** | | |
| --- | --- | --- |
|  | Mean | Standard Deviation |
| Overlapping Circles | 3.40 | 1.54 |
| Time-2 Perspective Taking | 5.30 | 2.24 |
| Time-2 Empathic Concern | 2.45 | 1.49 |
| Dictator Game Contributions | 3.21 | 2.16 |
| Trust Game 1^st^ Mover Contributions | 5.9 | 3.19 |
| Trust Game 2^nd^ Mover Contributions | 11.81 | 5.86 |
| CTG Time of Success  (in seconds) | 43.11 | 4.72 |
