# Supplementary figures and images for: Virtual reality perspective-taking increases cognitive empathy for specific others
Source: PLoS One. 2018 Aug 30;13(8):e0202442. doi: 10.1371/journal.pone.0202442 (PMC6116942; doi:10.1371/journal.pone.0202442)

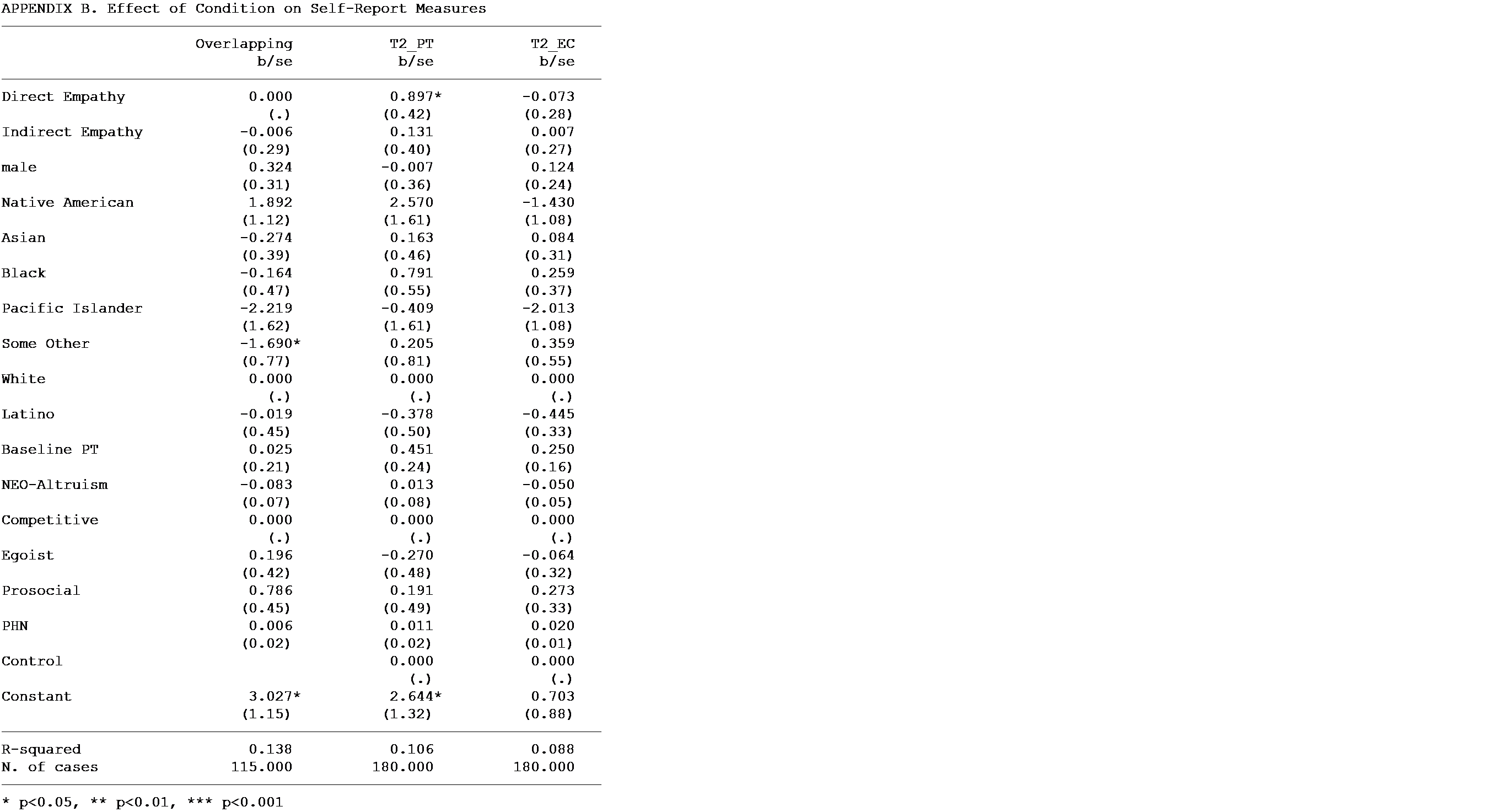

Supplement: S3 Table — Appendix C. Effect of condition on self-report measures with all controls. (TIF) [file pone.0202442.s003.tif]

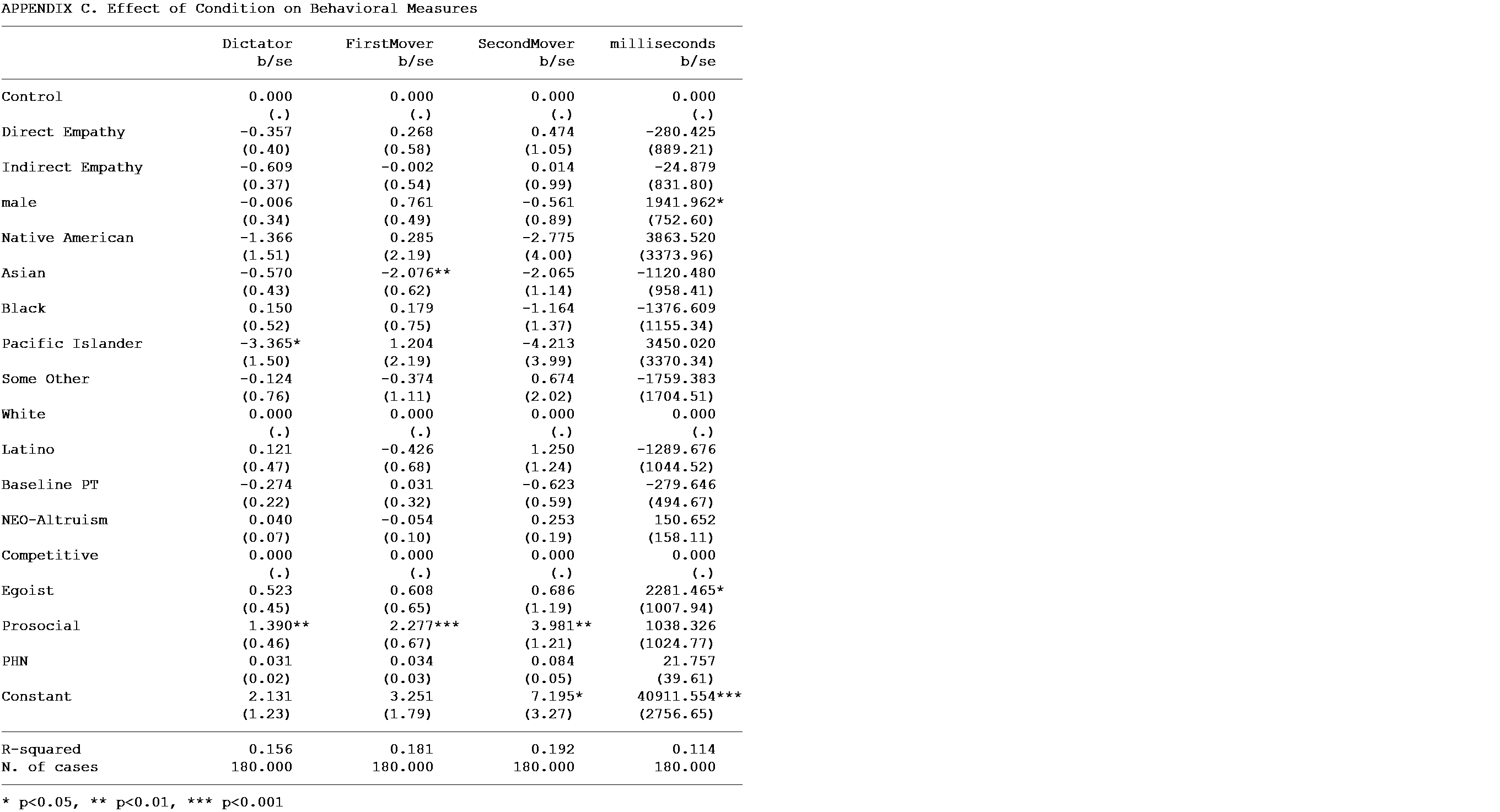

Supplement: S4 Table — Appendix D. Effect of condition on behavioral measures with all controls. (TIF) [file pone.0202442.s004.tif]
