# Supplementary material for: Virtual reality perspective-taking increases cognitive empathy for specific others
Source: PLoS One. 2018 Aug 30;13(8):e0202442. doi: 10.1371/journal.pone.0202442 (PMC6116942; doi:10.1371/journal.pone.0202442)
Supplement: S4 File — The script used by the researchers when being ran through the experiment. (DOCX) [file pone.0202442.s008.docx]

[PARTICIPANT ARRIVES IN LAB FOR PART TWO OF THE STUDY, VRPT]

“Hello and welcome to the Virtual Human Interaction Lab. Today you will be completing the second half of this two-part study investigating virtual reality technologies and their impact on people and society. If you’d please have a seat I’ll need you to review this consent form describing what we’ll be doing today.”

[RESEARCH ASSISTANT PROVIDES CONSENT FORM TO PARTICIPANT]

“During the study we will be recording both audio and video to ensure our data analysis is accurate. These recordings will be safely secured, only viewed by trained researchers, and destroyed after we have completed our data analysis. Your identity will be protected and none of our findings will be linked to you personally. If at any point in time you would like to take a break or remove yourself from the experiment, please let a researcher know immediately. We ask that you review this consent form, detailing that we will be recording both audio and video for the duration of your time in the lab. If you would like to continue please sign and date at the bottom of the page. Thank you.”

[RESEARCH ASSISTANT LEADS PARTICIPANT INTO THE LAB]

**EMPATHY CONDITIONS ONLY:**

“Please follow me into the lab and stand on the ‘X’ that is placed behind ‘VRPT’. During this part of the study you will be in an immersive virtual environment. You will be required to wear a head-mounted display and use two handheld devices to perform a series of tasks in the virtual environment, ‘A Day in the Life’. During this experiment you will be provided instructions in the form of audio voiceover and visual aids. It is important to listen and read everything closely. Additionally, you will be asked to perform a series of tasks using these handheld Vive controllers. “

[RESEARCHER SHOWS PARTICIPANT TRIGGER BUTTONS AND DEMONSTRATES USING THE HANDHELD CONTROLLER FOR SCENE 2]

“During the classroom scene, in order to advance to the next slide or return to a previous slide you must touch the corresponding arrow button and the trigger button on the Vive controller like this.”

[RESEARCHER DEMONSTRATES]

“A researcher will be in the room with you at all times to ensure your safety and make sure you do not fall or injure yourself. If at any point in time you would like to take a break or remove yourself from the experiment please let a researcher know immediately. Thank you.”

“You will be experiencing the life of [university name removed] student, [either ‘Steve’ or ‘James’, depending on condition]. We created a virtual world of what he described as a typical day based on the same questions you answered last week. In this experience you will first arrive in [either ‘Steve’s’ or ‘James’’, depending on condition] dorm room, then you will give a presentation in one of his classes, and then finally will visit the gym he works out in. You will find out what it is like to be [either ‘Steve’ or ‘James’, depending on condition], about his background, his family, his friends, and what he liked to do for fun.”

**CONTROL CONDITION ONLY:**

“Please follow me into the lab and stand on the ‘X’ that is placed behind ‘VRPT’. During this part of the study you will be in an immersive virtual environment. You will be required to wear a head-mounted display and use to handheld devices to experience a virtual representation of the physical lab room you are standing in right now. A researcher will be in the room with you at all times to ensure your safety and make sure you do not fall or injure yourself. If at any point in time you would like to take a break or remove yourself from the experiment please let a researcher know immediately. Thank you.”

**ALL CONDITIONS:**

“We will now get you equipped for the virtual reality experiment. We will put on the head-mounted display together and then I will place a controller in each of your hands. First, we will put the head-mounted display on your head. I ask that you hold the head-mounted display like this…”

[RESEARCHER DEMONSTRATES – HOLD HMD BY SIDES AT THE CORRECT AND COMFORTABLE EYE LEVEL]

“While you are holding the head-mounted display in position I will put the fastening straps around your head and slowly tighten the sides and top straps as needed. We want to make sure that the head-mounted display is fastened securely to your head, but is not too tight and not too loose, so your feedback as I tighten the strap and we make adjustments is appreciated. Then I will ask you to put your arms down by your sides while I retrieve the Vive controllers. I will then ask you to slightly raise your arms with your palms facing the ceiling. I will then place one Vive controller in each hand and have you put your hands back down by your side. Do you understand these instructions?”

[IF NO, ANSWER QUESTIONS AND/OR REPEAT INSTRUCTIONS AS NECESSARY]

[RESEARCHER EQUIPS PARTICIPANT WITH HMD AND VIVE CONTROLLERS]

“Now that you have been equipped with the appropriate devices, are you ready to begin the virtual reality portion of this study?”

[IF YES, PROCEED WITH EXPERIMENT. IF NO, ASK IF THERE’S AN ISSUE WITH HEADSET COMFORTABILITY, IF THEY ARE EXPERIENCING NAUSEA/DIZZINESS, ETC; ANSWER QUESTIONS, MAKE ADJUSTMENTS, AND/OR REPEAT INSTRUCTIONS AS NEEDED]

**POST-VR:**

“This concludes the virtual reality portion of today’s study. I am now going to ask you to raise your hands slightly so I can collect both controllers.”

[RESEARCH ASSISTANT COLLECTS CONTROLLERS]

“Next I am going to assist you in removing the head-mounted display

[RESEARCH ASSISTANT REMOVES HMD; START WALKING TOWARDS SURVEY ROOM]

“If you would please follow me to the survey room, we will now need you to complete a questionnaire and series of decision-making games. Once you have completed the last game please notify a researcher. You can begin the questionnaire once I have left the room.”

**POST-QUESTIONNAIRE:**

“Thank you for coming in today! The purpose of this immersive virtual reality experiment and subsequent questionnaire was to provide data on you, your life experiences, and your ability to coordinate with others based on virtual perspective-taking. Our goal was to investigate virtual reality technologies and their ability to influence cooperation and coordination through taking on the perspective of another individual. This experiment is still ongoing, so we ask that you not share any information you learned during your participation in this study with other people.”

“As part of this study we employed deception. In the prescreen, the questions we asked you specifically to create a potential virtual world of your life were not used. We built three worlds independent of anyone’s answers to the pre-screen, one of which you experienced today, but wanted you to believe that someone might embody you and experience a day in your life.”

**DIRECT EMPATHY CONDITION ONLY:**

“This means that the ‘A Day in the Life’ virtual experience you underwent, similarly, was created independent of anyone’s answers to the prescreen. [either ‘Steve’ or ‘James’, depending on condition] did not exist, and your answers weren’t paired with anyone else’s. We told you your answers would be paired with someone else’s so that you would behave as if you were interacting with a real person.”

**INDIRECT EMPATHY CONDITION ONLY:**

“This means that the ‘A Day in the Life’ virtual experience you underwent, similarly, was created independent of anyone’s answers to the prescreen. [either ‘Steve’ or ‘James’, depending on condition] did not exist. Also, your answers weren’t paired with anyone else’s. [either ‘Steve’ or ‘James’, depending on condition] also did not exist. We told you your answers would be paired with someone else’s so that you would behave as if you were interacting with a real person.”

**ALL CONDITIONS:**

“Also, compensation for you and all other participants was set to $50 regardless of your performance in the decision-making games. We told you that you would make between $5-$50 based on your performance to incentivize you to take all the tasks seriously. Do you have any questions regarding this study, any of the methods or deception we employed, or any of the data we are collecting?”

[ANSWER ALL QUESTIONS HERE]

“Our researchers have logged your questionnaire and you will now receive payment for this portion of the study. Please complete the following compensation form and sign/date here to confirm that you have received the payment. We keep this data solely for tax purposes in case we are audited and need to prove you are a real person who was paid for participating in this study. This information is securely stored in our lab and only accessible to trained researchers.”
